# Herbal Inhalation Therapy for Allergic Rhinitis: A Systematic Review and Meta-Analysis

**DOI:** 10.3390/ph18121877

**Published:** 2025-12-11

**Authors:** Sang-Song Shim, Jung-Eun Kil, Jiwon Park, Taehun Kim, Jungtae Leem, Beom-Joon Lee, Hee-Jae Jung, Kwan-Il Kim

**Affiliations:** 1 Department of Internal Medicine, College of Korean Medicine, Wonkwang University, 460, Iksan-daero, Sin-dong, Iksan 54538, Republic of Korea; trisss1011@gmail.com; 2 Department of Clinical Korean Medicine, Graduate School, College of Korean Medicine, Kyung Hee University, Seoul 02447, Republic of Korea; itsaqua188@naver.com (J.-E.K.); khujw5im@gmail.com (J.P.); rockandmineral@gmail.com (T.K.); franchisjun@naver.com (B.-J.L.); hanfish@khmc.or.kr (H.-J.J.); 3 Division of Allergy, Immune and Respiratory System, Department of Internal Medicine, College of Korean Medicine, Kyung Hee University, Kyung Hee University Medical Center, Seoul 02447, Republic of Korea; 4 Department of Diagnostics, College of Korean Medicine, Wonkwang University, Iksan 54538, Republic of Korea; julcho@naver.com; 5 Research Center of Traditional Korean Medicine, College of Korean Medicine, Wonkwang University, Iksan 54538, Republic of Korea; 6 Department of Il-won Integrated Medicine, Wonkwang University Korean Medicine Hospital, 895, Muwang-ro, Iksan 54538, Republic of Korea

**Keywords:** allergic rhinitis, herbal medicine, atomization inhalation, localized drug delivery, systematic review, meta-analysis

## Abstract

**Background:** Allergic rhinitis (AR) is a common immunological disorder characterized by nasal symptoms and impaired quality of life. Despite advances in pharmacotherapy, symptom control often remains inadequate. Herbal medicine atomized inhalation (HMAI), a modern adaptation of traditional East Asian fumigation, may offer an effective adjunct or alternative therapy. This systematic review and meta-analysis evaluated the clinical efficacy and safety of HMAI, emphasizing its role as a localized drug delivery system. **Methods:** Six databases were searched through 28 April 2025, for randomized controlled trials (RCTs) assessing HMAI for AR. Outcomes were pooled with random-effects meta-analysis; risk of bias and certainty of evidence were evaluated using Cochrane Risk of Bias 2.0 and GRADE. **Results:** Fourteen RCTs (*n* = 1606) met the inclusion criteria. HMAI significantly improved total effective rate compared with Western medicine (risk ratio = 1.20, 95% CI 1.09–1.32) and enhanced symptom relief when combined with Western or herbal treatments. However, the overall certainty of evidence ranged from moderate to very low due to methodological limitations across trials. **Conclusions**: HMAI may offer symptomatic benefits for patients with AR, particularly when used as an adjunct to existing therapies. Given the substantial variability and high risk of bias among included studies, these findings should be interpreted cautiously. Rigorous, placebo-controlled trials with standardized protocols are required to clarify the therapeutic role of HMAI in AR management.

## 1. Introduction

Allergic rhinitis (AR) is an immunoglobulin (Ig) E-mediated allergic inflammatory disease, characterized by rhinorrhea, nasal congestion, sneezing, and nasal itching [1]. Clinically, AR manifests as rhinorrhea, nasal congestion, sneezing, and nasal itching. This significantly impairs sleep quality and overall quality of life (QoL) [1,2,3]. AR affects an estimated 10–30% of adults and up to 40% of children worldwide [4]. Moreover, its prevalence has risen steadily in recent decades, along with a substantial socioeconomic burden [1,2,3]. Standard pharmacotherapies for AR, including oral H1-antihistamines, intranasal corticosteroids (INS), and leukotriene receptor antagonists, can provide symptomatic relief but often yield incomplete control and have notable adverse effects [5,6]. Antihistamines commonly induce sedation and xerostomia. Prolonged INS use can lead to nasal irritation or, rarely, systemic effects such as growth delay because of systemic absorption [7]. Allergen immunotherapy offers a disease-modifying approach but carries the risk of local or systemic allergic reactions and does not guarantee symptom resolution [8,9]. Moreover, conventional treatments generally do not address the underlying immune imbalances, and symptoms frequently recur after the cessation of therapy. This underscores the need for novel adjunct therapies that safely improve symptom control and target mucosal inflammation at its source [7].

Traditional East Asian medicine has long used herbal interventions for AR to correct presumed constitutional imbalances. Classical herbal formulations such as Xiao Qing Long Tang, Yu Ping Feng San, and Bu Zhong Yi Qi Tang have demonstrated effectiveness for AR in prior studies [10,11,12]. Fumigation therapy is an ancient treatment modality in which patients inhale warm vapors from heated or burned herbal preparations to alleviate respiratory and other ailments [13]. Historical records of herbal fumigation for nasal and airway conditions date back more than a millennium, and this practice is considered a precursor to modern intranasal herbal therapies [13,14]. Herbal fumigation can be viewed as a form of localized drug delivery that lays the foundation for modern herbal medicine atomized inhalation (HMAI). HMAI refers to the nebulization or vaporization of herbal decoctions such that patients can inhale the aerosolized extract directly through the nose [15]. By delivering therapeutic phytochemical compounds directly to the respiratory mucosa, HMAI concentrates the treatment effects at the disease site while minimizing systemic exposure. As a form of localized mucosal therapy, HMAI aligns with modern principles of targeted drug delivery for respiratory conditions. The intranasal inhalation route bypasses first-pass hepatic metabolism and reduces systemic drug distribution, thereby potentially lowering the risk of systemic side effects while achieving rapid and direct relief at the nasal mucosa [16]. Given the drawbacks of existing AR treatments, there is a growing clinical interest in whether this modernized inhalation therapy, derived from traditional fumigation, can enhance AR management outcomes. Early clinical reports, mostly from East Asia, suggest that HMAI, used either alone or as an add-on to conventional medicine, can improve nasal symptoms and modulate immune markers of AR, with few adverse events [17,18]. For example, certain herbal inhalation treatments have been associated with reductions in serum IgE and interleukin (IL)-5 levels along with symptom improvement, indicating potential immunomodulatory effects [17]. These preliminary findings are encouraging, but there remains an evidence gap regarding HMAI’s efficacy and safety according to rigorous international standards. To date, many studies on HMAI for AR have been small or lacked placebo controls. Furthermore, there is heterogeneity in the herbal formulations and outcome measures.

In light of this promising yet inconclusive evidence, we aimed to evaluate the efficacy and safety of HMAI in patients diagnosed with allergic rhinitis. To our knowledge, no prior review has specifically synthesized randomized controlled trials (RCTs) of herbal medicine HMAI as an inhaled, mucosa-targeted intervention for AR, nor comprehensively appraised the certainty of evidence—including immunological biomarkers and safety—using the risk of bias (RoB) 2 and The Grading of Recommendations Assessment, Development, and Evaluation (GRADE). Our review was therefore designed to address this evidence gap. The experimental groups included regimens in which HMAI was used as monotherapy or in combination with other medicine, whereas the control groups included regimens with Western pharmacotherapy alone or in combination with other medicine for AR, excluding HMAI treatment. We focused on patient-important outcomes, primarily the total effective rate (TER, a composite measure of symptom improvement as defined in the trials), objective immunological indices (e.g., total IgE and inflammatory cytokines), and reported adverse events. By synthesizing the current evidence, we aimed to clarify the therapeutic value of HMAI, examine its potential immunomodulatory effects, and assess whether this intervention could be integrated into conventional AR management. This review also aimed to identify the limitations of existing research and guide future studies on standardized HMAI protocols for allergic rhinitis.

## 2. Materials and Methods

This systematic review and meta-analysis conformed to the Preferred Reporting Items for Systematic Reviews and Meta-Analyses (PRISMA) guidelines [19], with the completed PRISMA checklist provided in Supplementary File S1. The study protocol was registered in PROSPERO (ID: CRD42023476037).

### 2.1. Inclusion and Exclusion Criteria

#### 2.1.1. Study Types

Only RCTs mentioning “randomization” were included. No restrictions were applied regarding the treatment duration or clinical settings. Non-RCTs in humans (e.g., case reports and review articles) and animal experimental studies were excluded.

#### 2.1.2. Participants

Patients diagnosed with AR were included in the study. No restrictions were applied regarding disease stage, age, sex, race, country, hospitalization, or outpatient status.

#### 2.1.3. Intervention Types and Controls

The treatment group included patients who received HMAI, defined as the inhalation of steam generated by the application of heat or ultrasonic energy to single or multiple herbal decoctions using a specialized device, as the primary intervention. The control group included patients who received the standard treatment for AR, excluding HMAI treatment.

#### 2.1.4. Outcome Measures

The primary outcome was the TER. TER is expressed using the four categories “cured”, “markedly effective”, “effective”, and “ineffective”, and was dichotomized into effective and ineffective. Secondary outcomes included immune factors and adverse events.

### 2.2. Literature Searches

Six electronic databases (English, Korean, and Chinese), MEDLINE (via PubMed), EMBASE (via Elsevier), Cochrane Central Register of Controlled Trials (CENTRAL), Korean Studies Information Service System (KISS), Oriental Medicine Advanced Searching Integrated System (OASIS), and China National Knowledge Infrastructure (CNKI), were searched up to 28 April 2025. To increase search sensitivity in the literature search process, the following HMAI-related terms were used: “rhinitis,” “fumigation,” “aromatherapy,” “nebulizer,” “inhalation,” and “atomization.” No country or language restrictions were imposed. Additionally, when translating “fumigation therapy” into English, it was noted that “fumigation” may include the commonly understood concept of disinfection, and that “aromatherapy” may be confused with traditional aromatherapy, whereas “nebulizer” could limit the method of fumigation therapy to the use of conventional nebulizers. Therefore, the term “herbal medicine atomization inhalation” was consistently used. The detailed search terms and strategies for each database are provided in Supplementary File S2.

### 2.3. Study Selection

Two authors (SSS and JEK) independently screened the relevant literature. The search results were compared to ensure that no studies were omitted. Disagreements were resolved consensually, or a third author (JL or KIK) was consulted to decide on the final inclusion. The selected literature was organized and managed using EndNote software (version 20.3; Clarivate Analytics, Philadelphia, PA, USA), which facilitated deduplication and streamlined the reference organization. Titles and abstracts were first reviewed, to exclude studies irrelevant to the study design, intervention, or target population. The full texts of the resulting studies were assessed for eligibility based on the inclusion criteria. After reviewing titles, abstracts, and full texts, only studies that included HMAI treatments were selected.

### 2.4. Data Extraction

Two independent researchers (SSS and JEK) extracted the data, which were organized using Microsoft Excel (version 2019; Microsoft Corp., Redmond, WA, USA). The following information was extracted: author, title, publication year, language, study type, number of participants, age, intervention (method, medicine composition, dosage, and formulation), treatment duration, frequency, outcome measures, and results.

### 2.5. Risk of Bias Assessment

RoB in the included RCTs was assessed using the revised Cochrane Risk of Bias 2.0 (RoB 2.0) tool [20]. This tool evaluates five bias domains: (1) bias arising from the randomization process, (2) bias because of deviations from intended interventions, (3) bias because of missing outcome data, (4) bias in the measurement of the outcome, and (5) bias in the selection of reported results. Each domain was judged as having a “low RoB,” “some concerns,” or a “high risk of bias.” Based on domain-level judgments, an overall risk of bias judgment was derived for each study. Two independent reviewers (SSS and JEK) assessed RoB. Disagreements were resolved through discussion, and a third reviewer (JL or KIK) was consulted when necessary.

### 2.6. Data Analysis

#### 2.6.1. Quantitative Synthesis

Data were synthesized using Review Manager software (RevMan version 5.4.1; Cochrane Collaboration, Copenhagen, Denmark). An analytical framework was developed to ascertain suitable studies for each synthesis. This involved tabulating the study intervention characteristics and comparing them with the planned groups for each synthesis. To reduce between-study heterogeneity, the analyses were grouped based on study design. Owing to the inherent heterogeneity of fumigation therapy, which is typical in Traditional East Asian Medicine treatments, and the design characteristics of the included studies, a random-effects model (REM) rather than a fixed-effects model (FEM) was primarily applied in the meta-analysis. Meta-analyses were performed within groups when two or more studies reported the same outcome measure and were visualized using forest plots.

Considering the clinical heterogeneity caused by variations in interventions across studies, effect sizes were calculated using the REM and classified as small (0.20–0.50), medium (0.50–0.80), or large (>0.80), based on Cohen’s d. Dichotomous data (effective rate) were presented as risk ratios (RR) with 95% confidence intervals (CI). Heterogeneity within the individual analyses was assessed using the *I*^2^ statistic. The statistical significance of effect sizes was determined using overall effect testing or a 95% CI. The statistical significance level set at 5%. Adverse effects were reported by frequency without statistical analysis.

#### 2.6.2. Sensitivity, Subgroup, and Publication Bias Analyses

Sensitivity analysis was conducted when a sufficient number of studies (at least 10 per study design) were available. Studies with high RoB were excluded to assess whether low-quality studies influenced the overall results. Additionally, a subgroup analysis was conducted by excluding studies from specific countries to evaluate whether geographical variations influenced the results. Publication bias was assessed when a sufficient number of studies (at least 10 per study design) were available, qualitatively and visually using a funnel plot and quantitatively using Egger’s test. If the *p*-value of the Egger’s test was <0.10, publication bias was considered to be present.

#### 2.6.3. Certainty of Evidence

GRADE method was used to assess the certainty of evidence. It was classified as “high,” “moderate,” “low,” or “very low” using the online version of GRADEpro GDT software (https://www.gradepro.org/ (accessed on 1 May 2025)). “High” certainty indicated strong confidence that the estimated effect closely resembled the actual effect, whereas “very low” certainty suggested substantial discrepancy between the estimated and actual effects.

## 3. Results

### 3.1. Study Selection

**Figure 1:**
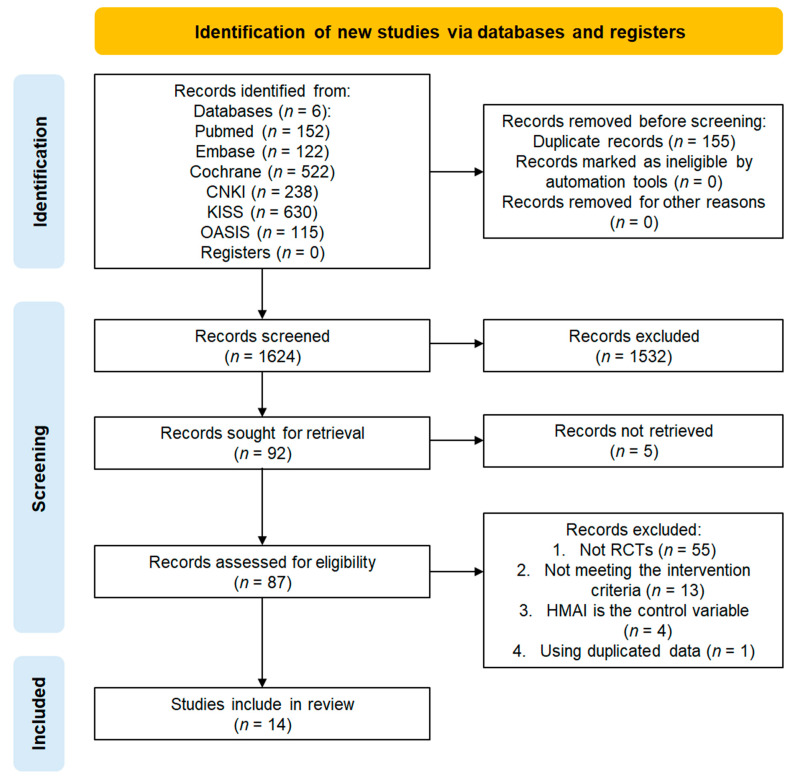
Study selection flowchart.

A total of 1779 studies were identified on MEDLINE (*n* = 152), EMBASE (*n* = 122), CENTRAL (*n* = 522), CNKI (*n* = 238), KISS (*n* = 630), and OASIS (*n* = 115). After deduplicating 155 studies, 532 studies that did not meet the selection criteria after title and abstract screening and five studies that could not be accessed for full-text review were excluded. Among the 87 studies selected for full-text evaluation, 73 were excluded for the following reasons: they were not RCTs (*n* = 55), did not meet the intervention criteria (*n* = 13), and inclusion of HMAI in the control group (*n* = 4), and duplicates (*n* = 1). Consequently, 14 studies were included in the final analysis, all of which were meta-analyzed. The study selection process is illustrated in the PRISMA flow diagram (Figure 1) [21].

### 3.2. Study Characteristics

All included studies were conducted between 2001 and 2022 and were published in Chinese. Their detailed characteristics are summarized in Table 1. A total of 1606 participants (patients diagnosed with AR) were enrolled, with 60–120 participants per study, and no dropouts reported. The participants’ ages, disease durations, and treatment periods ranged from 1 to 74 years, 1 month to 17 years, and 6 days to 2 months, respectively.

The treatment and control groups were broadly categorized into four groups. The interventions in Groups 1 and 2 were designed as add-on therapies, whereas those in Group 3 included only the HMAI treatment. Five studies [22,23,24,25,26] compared a treatment group that received both Western medicine (WM) and HMAI, with a control group that received only WM (Group 1, WM + HMAI vs. WM). Three studies [27,28,29] compared a treatment group that received HM combined with HMAI to a control group that received only HM (Group 2, HM + HMAI vs. HM). Five studies [30,31,32,33,34] compared a treatment group that received HMAI with a control group that received WM (group 3, HMAI vs. WM). One study [35] compared a treatment group that received comprehensive HM, including HMAI, with a control group that received comprehensive WM (Group 4, comprehensive HM treatment vs. comprehensive WM treatment).

Fourteen studies evaluated the effectiveness of HMAI treatment. Five of these studies assessed clinical symptom scores, two evaluated immune indicators, two assessed Traditional Chinese Medicine (TCM) syndrome scores, and one evaluated QoL using the Rhinoconjunctivitis QoL Questionnaire (RQLQ). Additionally, one study examined the relationship between treatment effectiveness and TCM syndrome type, one assessed physical sign scores, one evaluated complications, and one assessed the recurrence rate (Table 2).

The most frequently used herbs in the HMAI treatment group were *Xanthium strumarium* L. (Xanthii Fructus, 10 times) and *Magnolia denudate* Desr. (Magnoliae Flos; 10×), followed by *Angelica dahurica* (Hoffm.) Benth. & Hook.f. ex Franch. & Sav. (Angelicae Dahuricae Radix; 9 times), *Mentha canadensis* L. (Menthae Herba; six times), *Glycyrrhiza uralensis* Fisch. ex DC. (Glycyrrhizae Radix et Rhizoma; six times), and *Astragalus mongholicus* Bunge (Astragali Radix; five times). The volume of the liquid used in HMAI was 10–500 mL. The duration of HMAI treatment sessions was 10–30 min, either once or twice daily, or once every 2 days. The formulations, Latin names of the herbs, and methods of HMAI treatment used in each study are presented in Table 3, and the frequently used herbs are listed in Table 4.

### 3.3. RoB of Included Studies

**Figure 2:**
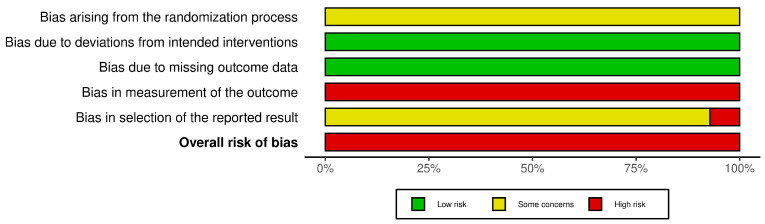
Risk of bias assessment results: Risk of bias summary.

**Figure 3:**
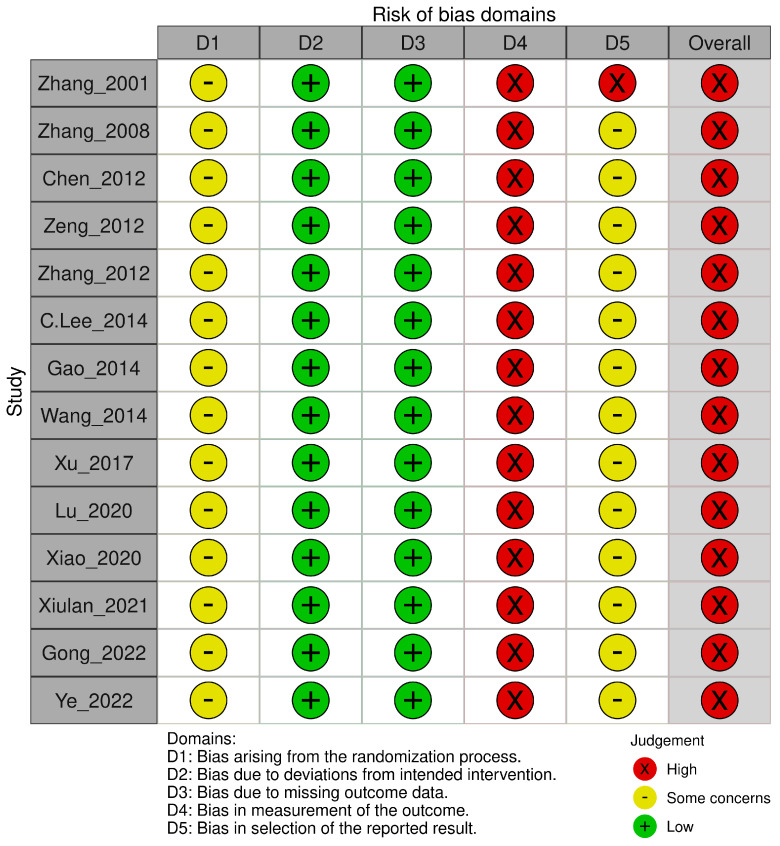
Risk of bias assessment results: Risk of bias graph [22,23,24,25,26,27,28,29,30,31,32,33,34,35].

The Cochrane RoB 2.0 assessment revealed methodological concerns across all 14 included RCTs (Figure 2 and Figure 3). Most studies demonstrated concerns or high risks across multiple domains. For the randomization process, all studies reported random allocation and balanced baselines but failed to adequately describe the allocation concealment procedures. Deviations from intended interventions presented a low risk across all trials, as no protocol deviations or withdrawals were reported, despite the inability to blind participants owing to the inherent characteristics of the HMAI intervention. The missing outcome data showed a consistently low risk with complete reporting in all studies. However, measurement of outcomes represented the most significant limitation, with all trials receiving high risk ratings because of the impossibility of blinded outcome assessment. This was due to the inherent characteristics of HMAI, which make it difficult to establish credible placebo controls, and most reported outcomes were effectiveness rates evaluated based on patient symptom reports. For the selection of reported results, no studies provided publicly available protocols or pre-specified analysis plans. This resulted in some concerns for 13 studies and a high risk for one study [28] because of insufficient statistical methodology details. Overall, all 14 trials were classified as having a high risk of bias, primarily driven by unblinded outcome assessments, necessitating a cautious interpretation of the pooled efficacy estimates.

### 3.4. Effects of Interventions by Group and Outcome Measures

**Figure 4:**
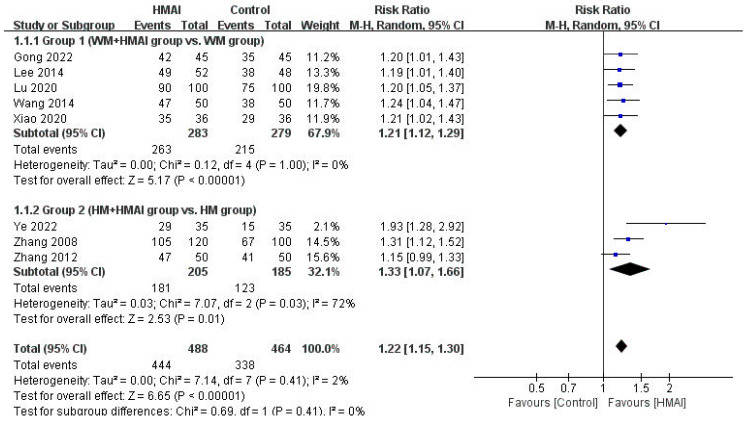
Forest plot of the meta-analysis of the total effective rate of HMAI as add-on therapy [22,23,24,25,26,27,28,29].

**Figure 5:**
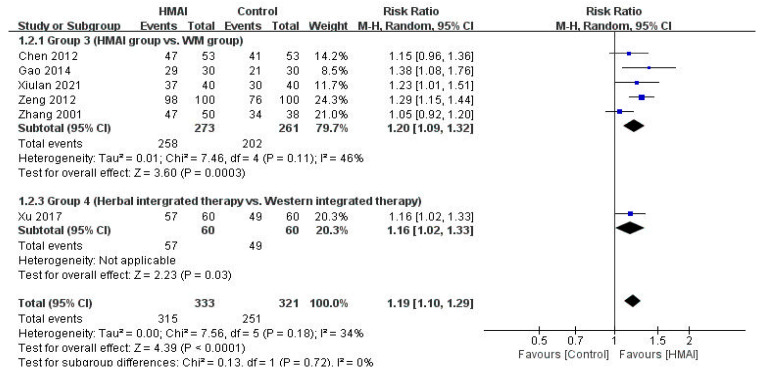
Forest plot of the meta-analysis of the total effective rate of HMAI versus [30,31,32,33,34,35].

The effectiveness of the interventions in the four groups, compared by quantitatively synthesizing studies using similar intervention methods and outcome measurements, is presented in a forest plot (Figure 4 and Figure 5). Sensitivity, subgroup, and publication bias analyses were not performed because of the insufficient number of available studies.

#### 3.4.1. Add-On Therapy Design

##### Group 1 (WM + HMAI vs. WM)

Regarding TER, the meta-analysis of the five studies in Group 1 revealed a significant improvement in the WM + HMAI group (RR = 1.21, 95% CI: 1.12–1.29), with no observed heterogeneity (*I*^2^ = 0%) (Figure 4). Other secondary outcome measures and immune indicators varied considerably in the interval and measurement tools used for the meta-analysis and were qualitatively described. One study [23] reported immune markers, revealing significantly lower IL-6, IL-8, and tumor necrosis factor (TNF)-α levels in the treatment group than in the control group (*p* < 0.05).

##### Group 2 (HM + HMAI vs. HM)

Regarding TER, the meta-analysis of three studies in Group 2 revealed a significant improvement in the HM + HMAI group (RR = 1.33, 95% CI: 1.07–1.66), with moderate heterogeneity (*I*^2^ = 72%) (Figure 4). None of the studies in the HM + HMAI group assessed immune indicators or adverse events.

#### 3.4.2. Monotherapy Design

##### Group 3 (HMAI vs. WM)

Regarding TER, the meta-analysis of the five studies in Group 3 revealed significant benefits in the HMAI group (RR = 1.20, 95% CI: 1.09–1.32), with moderate heterogeneity (*I*^2^ = 46%) (Figure 5). Only one study [31] assessed immune indicators and reported significantly lower IL-4 and sIgE levels in the HMAI group than in the WM group (*p* < 0.05).

##### Group 4 (Herbal Integrated Therapy Group vs. Western Integrated Therapy Group)

The herbal integrated therapy group had significantly higher TER (RR = 1.16, 95% CI: 1.02–1.33, *n* = 120) (Figure 5), significantly higher IL-12 and TNF-γ levels, and significantly lower IgE levels than the Western integrated therapy group (*p* < 0.05) [35].

### 3.5. Adverse Events and Safety

Only one study [24] in Group 1 reported adverse events such as general fatigue, localized infections, nausea, and allergic skin reactions. While 13 cases were reported in the control group, only 5 cases were reported in the treatment group, indicating significantly fewer adverse events in the treatment group compared with the control group (*p* < 0.05).

### 3.6. Certainty of Evidence

The certainty of evidence across all groups was downgraded because of high RoB. The certainty of evidence was rated as “moderate” for TER in Group 1, “low” in Group 2, and “very low” in Group 3. Inconsistencies were noted in Groups 2 and 3, as the meta-analysis results suggested potential heterogeneity. In Group 3, the certainty of evidence in the treatment group was rated as “serious” for indirectness owing to variations in specific methods, such as consuming the HMAI solution after inhalation (Table 4).

## 4. Discussion

HMAI is a modern adaptation of traditional herbal fumigation therapy that is analogous to conventional inhalation treatments for respiratory conditions. This review provides the first comprehensive evaluation of HMAI for allergic rhinitis across multiple studies. We included patients diagnosed with allergic rhinitis, with intervention groups receiving regimens that incorporated HMAI and control groups receiving standard treatments for allergic rhinitis that did not include HMAI. The primary outcome TER, and to reduce clinical heterogeneity, we stratified the meta-analysis by study design, separating trials in which HMAI was used as an add-on therapy from those in which it was used as monotherapy.

In this study, we conducted a systematic review and meta-analysis of RCTs to assess the efficacy and safety of HMAI in AR management. Fourteen RCTs [22,23,24,25,26,27,28,29,30,31,32,33,34,35], including 1606 patients, were identified from six domestic and international databases, regardless of publication language, and grouped into four categories based on study design. After excluding Group 4, which included only one study, a meta-analysis was conducted for Groups 1–3, with TER as the primary outcome. The results revealed the positive effects of HMAI treatment on improving AR symptoms: Group 1 (WM + HMAI vs. WM) (RR = 1.21, 95% CI: 1.12–1.29, *I*^2^ = 0%, REM, *N* = 5, *n* = 562), Group 2 (HM + HMAI vs. HM) (RR = 1.33, 95% CI: 1.07–1.66, *I*^2^ = 72%, REM, *N* = 3, *n* = 390), and Group 3 (HMAI vs. WM) (RR = 1.20, 95% CI: 1.09–1.16, *I*^2^ = 46%, REM, *N* = 5, *n* = 534). However, the overall RoB was rated as high, and the certainty of evidence ranged from “moderate” to “very low.” Therefore, the results should be interpreted with caution. Importantly, our findings should not be interpreted as evidence that HMAI is superior to established pharmacotherapy; rather, the main clinical focus of this review is whether HMAI provides incremental benefit when used as an adjunct to standard Western or oral herbal regimens.

The results of Groups 1 and 2, in which HMAI was an add-on to either WM or HM, revealed that combining HMAI with conventional Western or herbal treatments was more effective for AR than these treatments alone. This suggests that incorporating HMAI into conventional therapeutic strategies may further improve AR symptoms.

In Group 3, HMAI monotherapy was more effective than WM alone. However, cautious interpretation is needed owing to the small number of included studies (*N* = 5) and the “very low” certainty of evidence. Nonetheless, for pediatric patients who are more susceptible to the adverse effects of WM, HMAI therapy could serve as a viable option, either as a stand-alone or add-on treatment. Notably, two studies in Group 3 [25,26] focused exclusively on pediatric patients and reported that HMAI was more effective than conventional WM nebulizer treatments.

A study on the effectiveness of HM for AR found that HM was more effective than loratadine (RR = 1.23, 95% CI: 1.16–1.22, *I*^2^ = 30%, FEM, *N* = 11, *n* = 956) [36]. This effect size was comparable to that found in Group 3 (HMAI vs. WM) in the present study. Additionally, in Group 2 (HM + HMAI vs. WM), the effect size was large, suggesting that the combined approach of HM and HMAI may be even more effective for AR than HM alone. Although other studies addressing AR have evaluated additional outcomes, such as the total nasal symptom score or RQLQ, a meta-analysis across these measures could not be conducted in the present study because of the heterogeneity of the outcome measures in the included studies. Consequently, direct comparisons of the effect sizes with those of other studies are limited. Therefore, future RCTs should be conducted using objective and standardized evaluation tools.

In the included studies, the most frequently used herbs in the HMAI treatment of AR were Xanthii Fructus and Magnoliae Flos, followed by Angelicae Dahuricae Radix, Menthae Herba, Glycyrrhizae Radix et Rhizoma, and Astragali Radix. Several studies have investigated the effects of these herbs. Xanthii Fructus inhibits histamine release from peritoneal mast cells and suppresses systemic anaphylaxis, in addition to IgE-mediated passive cutaneous anaphylaxis (PCA) reactions [37]. Magnoliae Flos exhibits anti-inflammatory properties [38], suppresses auricular edema responses, and IgE-induced PCA reactions, and inhibits histamine release from peritoneal mast cells [39]. Angelicae Dahuricae Radix reduces the total number of inflammatory cells more effectively than montelukast, a leukotriene antagonist used for AR, and demonstrates anti-asthmatic effects by significantly suppressing IL-4 and IL-5 production in bronchoalveolar lavage fluid [40]. Menthae Herba exerts antimicrobial effects by inhibiting the growth of *Staphylococcus aureus*, *Shigella dysenteriae*, and *Escherichia coli* [41]. Additionally, it relaxes acetylcholine-induced vascular contractions [42]. Astragaloside IV, extracted from Astragali Radix, reduces histamine-induced inflammatory responses in animal models by partially blocking the NF-κB signaling pathway, rendering it potentially effective against AR [43]. Taken together, these findings indicate that the core herbs most frequently used in HMAI formulations for AR may exert anti-inflammatory and anti-allergic effects by inhibiting mast-cell degranulation and histamine release, modulating Th2-skewed immune responses, and suppressing NF-κB–related inflammatory signalling pathways, which is mechanistically consistent with the improvements in TER observed in our meta-analysis and with the direction of immunological changes (e.g., IgE and cytokines) reported in a limited subset of included trials [37,38,39,40,41,42,43].

In clinical practice, the most commonly prescribed herbs for AR are Xanthii Fructus, Magnoliae Flos, Angelicae Dahuricae Radix, Menthae Herba, Astragali Radix, and Saposhnikoviae Radix [44]. This prescription pattern closely mirrors the findings of the present study (see Additional File 4), indicating strong concordance between real-world clinical practice and the present study’s dataset. Pharmacognostic reviews further indicate that most of these herbs enter the Lung meridian and either dispel wind pathogens or tonify Lung qi [45].

Specifically, Xanthii Fructus, Magnoliae Flos, and Angelicae Dahuricae Radix are pungent and warm exterior-releasing herbs that rapidly eliminate exogenous wind-cold and open nasal orifices. Moreover, these herbs are key components of traditional formulas such as Xinyi-San and Cang-er-Zi-San, recorded in the “Bang-yak Hap-pyeon”. While Menthae Herba disperses wind-heat and helps relieve sneezing and nasal congestion, Astragali Radix, a sweet-warm qi-tonifying herb, strengthens Lung and Spleen qi, reinforces the body’s defensive barrier, wei-qi, and modulates allergic responses [46]. Based on these pharmacological and traditional properties, pattern-based combinations of these herbs delivered via HMAI are expected to further enhance the therapeutic outcomes in AR management.

Nevertheless, this study has several limitations. First, all the included studies were conducted in China, presenting a geographical limitation. Second, the between-study heterogeneity due to the varied treatment types used in the control groups warrants cautious interpretation of the results. Additionally, none of the studies used a placebo control group, limiting their ability to provide strong evidence for the effectiveness of HMAI treatment alone. Third, although subgroup analyses based on study design reduced heterogeneity, the number of studies included in each meta-analysis was relatively small (3–5 studies per analysis). Fourth, only one study reported adverse events in which the intervention group received both HMAI treatment and acupoint herbal plaster therapy; hence, the safety of HMAI treatment alone could not be conclusively determined. In addition, safety and complications were not prospectively monitored or reported using standardized and validated definitions across trials, and most studies provided only brief statements (e.g., “no adverse events”) or no safety information at all; thus, the current evidence is insufficient to draw firm conclusions regarding the safety of HMAI (particularly for longer-term use). Fifth, the specific methods of HMAI treatment varied across the studies. In some cases, the herbal decoction was reheated to allow steam inhalation, whereas other methods involved ultrasonic nebulization. Sixth, the number of herbal medicines used per session and the duration, frequency, and time spent per treatment varied considerably across the studies. Seventh, the included trials encompassed patients across a broad age range. Children and adults differ in immune responses, nasal anatomy, and mucosal absorption characteristics, and their therapeutic responses may therefore also differ. In order to include all available RCTs, we did not impose age restrictions in the present review; however, this decision may have introduced additional clinical heterogeneity. Eighth, the lack of uniform outcome measures among the studies limited the comparison of effect sizes in the present study. TER is a composite outcome that is widely used in Chinese-language TCM clinical trials but is not globally standardized, and variability in how the categories (“cured”, “markedly effective”, “effective”) are operationalized across studies may introduce heterogeneity and limit the comparability and interpretability of our pooled estimates. Future clinical trials with consistent outcome measures, such as total nasal symptom score, RQLQ, and measures of adverse events for safety, are needed to robustly evaluate AR symptoms and QoL improvements with HMAI treatment. As HMAI is effective when added to conventional pharmacotherapy or other herbal treatments, further research on standardized HMAI protocols is essential to improve its practical application in AR treatment, in clinical settings.

Because HMAI delivers warmed vapor or aerosolized decoctions directly to the airway mucosa, inhalation-specific safety issues warrant explicit attention. In particular, steam-based delivery may pose a risk of thermal injury (scalds have been reported, especially in children), underscoring the need to control preparation temperature, inhalation distance, and supervision [47]. In addition, reusable nebulizer components can become microbially contaminated and may act as reservoirs delivering pathogens if cleaning and disinfection are inadequate; therefore, future HMAI trials should standardize device specifications and hygiene protocols and incorporate prospective monitoring of respiratory adverse events [48]. Given reported concerns regarding contaminants in herbal medicines (e.g., heavy metals) and potentially toxic constituents in certain botanical materials (e.g., aristolochic acids), chemical characterization, quality control, and inhalation-focused toxicological assessment should be incorporated to support a robust safety evaluation of aerosolized herbal preparations [49].

A notable strength of this study is that, to the best of our knowledge, it is the first systematic review to focus solely on the effects of HMAI treatment on AR. Moreover, it followed the PRISMA guidelines to provide more comprehensive and reliable conclusions on the effects of HMAI treatment for AR. Studies from both domestic and international databases, regardless of publication language or study design, were included to analyze as many studies as possible. This increased the external validity of the research and rendered the results more generalizable. These strengths also lead to discussions on future therapeutic applications. Furthermore, given the favorable efficacy and safety profiles of the herbal formulations used in the included HMAI interventions, these findings provide a valuable foundation for the future development of natural product-based nebulized therapies. Such formulations could serve as modernized inhalation agents for inflammatory airway diseases, such as allergic rhinitis and asthma, offering potential advantages in biocompatibility, localized delivery, and reduced systemic toxicity. This highlights the translational relevance of traditional fumigation therapies in modern pharmaceutical innovation.

## 5. Conclusions

HMAI demonstrated improvements in TER across several AR treatment comparisons in the included RCTs, suggesting potential symptomatic benefits, particularly when used as an adjunct to conventional therapy. However, because the overall risk of bias was high and the certainty of evidence ranged from moderate to very low, these findings should be interpreted with caution. The heterogeneity of interventions, limited reporting of safety outcomes, and lack of standardized evaluation tools further restrict the strength of the conclusions. Well-designed, placebo-controlled clinical trials using standardized HMAI protocols and validated outcome measures are needed to more definitively determine its efficacy and safety.

## Figures and Tables

**Table 1:** Characteristics of the included studies.

Study	Sample Size ((A):(B))	Mean Age (Years) (Age Range)	Sex (M:F)	Duration of Illness (Duration Range	(A) Treatment Intervention	(B) Control Intervention	Treatment Period (Days)	Clinical Outcome Measure
Group 1. WM + HMAI vs. WM								
Xiao et al. (2020) [22]	72 (36:36)	(A) 40.3 ± 1.3 (B) 41.3 ± 1.2	(A) 17:19 (B) 16:20	(A) 4.5 ± 1.32 (0.8–9) years (B) 4.6 ± 1.3 (0.6–9) years	(B) (1) (2) HMAI (Xanthium powder combined with mahuang decoction)	(1) Levocetirizine hydrochloride dispersible tablets 5 mg PO qd	30	1. Effective rate 2. Clinical symptom score 3. Nasal sign
Gong et al. (2022) [23]	90 (45:45)	(A) 5.44 ± 1.97 (B) 5.16 ± 1.71	(A) 26:19 (B) 25:20	(A) 2.11 ± 0.71 years (B) 2.32 ± 0.75 years	(B) (1)–(3) (4) HMAI (Xinyisan decoction)	(1) Desloratadine dry suspension 1.25–2.5 mg PO (2) Montelukast sodium chewable tablets 4 mg PO (3) Mometasone furoate nasal spray 50 mg	14	1. Effective rate 2. TCM symptom scores 3. Laboratory indicators (IL-6/IL-8/TNF-α)
Lu et al. (2020) [24]	100 (40:60)	(A) 44.6 ± 6.7 (25–74) (B) 42.6 ± 6.4 (24–71)	(A) 40:60 (B) 42:58	(A) 5.7 ± 2.6 (1–12) years (B) 5.6 ± 2.4 (1–13) years	(B) (1) and (2) (3) HMAI with acupoint application	(1) Triamcinolone nasal spray (2) Loratadine 10 mg PO	15	1. Effective rate 2. RQLQ 3. Symptom Score 4. Adverse events
Wang et al. (2014) [25]	100 (50:50)	(A) 31.33 ± 10.06 (B) 32.4 ± 11.97	(A) 24:26 (B) 30:20	(A) 16.87 ± 12.55 years (B) 15.57 ± 11.89 years	(B) (1)–(2) (3) HMAI (Self-formulated Cangzhi combination)	(1) Loratadine 10 mg PO (2) Budesonide suspension ultrasonic nebulization therapy/Budesonide spray	60	1. Effective rate
Lee et al. (2014) [26]	100 (52:48)	(A) 48.3 (18–78) (B) 46.7 (17–77)	(A) 28:26 (B) 28:20	(A) 1–15 years (B) 0.8–14	(B) (1) (2) HMAI (Xanthium powder) 30 min 14 days * If symptoms are severe: Sesame oil with *Xanthium sibiricum* nasal drops 7 days	(1) Loratadine 10 mg PO 14 days (2) Budesonide nasal spray 30 days	(A) 14 (B) 30	1. Effective rate
Group 2. HM + HMAI vs. HM								
Ye et al. (2022) [27]	70 (35:35)	(A) 35.78 ± 11.13 (B) 38.43 ± 11.09	(A) 20:15 (B) 22:13	(A) 6 (3–11) years (B) 7 (2–11) years	(B) (1) (2) HMAI (Xanthium powder)	(1) Wenfei Zhiliu Dan PO	7	1. Symptom score 2. Effective rate
Zhang et al. (2012) [28]	100 (50:50)	(A) 4–15 (B) 4–13	(A) 29:21 (B) 31:19	(A) 3 months–5 years (B) 3 months–6 years	(B) (1) (2) HMAI (Xinjiu nebulization solution)	(1) OnPaiJiRyuDan or Samryungbeakchul-san or Joaguihwan (or Youguihwan) decoction PO	7	1. Effective rate 2. Relationship between the effectiveness in the treatment group and the TCM syndrome types
Zhang et al. (2008) [29]	220 (120:100)	(A) 28 (13–70) (B) 30 (5–70)	(A) 43:77 (B) 38:62	(A) 4.3 (3–17) years (B) 3.9 (2–16) years	(B) (1) (2) YuPingFengSan combined with Kyejitang and Xanthium powder HMAI	(1) YuPingFengSan + Kyejitang + Xanthium powder decoction PO	30	1. Effective rate
Group 3. HMAI vs. WM								
Xiulan (2021) [30]	80 (40:40)	40 (14–66)	38:42	1 month–8 years	(1) Siwei Tumuxiang Decoction HMAI + P.O	(1) Budesonide nebulizer	21	1. Symptom score 2. Effective rate
Gao (2014) [31]	60 (30:30)	2–12	35:25	NR	(1) HMAI (Jiegeng Yuanshen Tang combined with Yupingfeng San)	(1) Loratadine 5 mg/day atomization inhalation	7	1. Laboratory indicators (ρIL-4, sIgE) 2. Effective rate
Zeng et al. (2012) [32]	100 (45:45)	(A) 1–14 (B) 1–14	(A) 53/47 (B) 52/48	NR	(1) HMAI (Cangxin liquid)	(1) (0.9% Uranium chloride 20 mL + gentamicin 80,000 U + dexamethasone 2 mg) Ultrasonic atomization inhalation	21	1. Effective rate
Chen (2012) [33]	106 (53:53)	(A) 33.20 ± 9.56 (19–57) (B) 31.45 ± 9.12 (18–56)	(A) 26/27 (B) 28/25	(A) (1.5–13) years (B) (1–11) years	(1) Xiaoqinglongtang HMAI + P.O	(1) Loratadine 10 mg PO	21	1. Symptom score 2. Physical sign score 3. Effective rate 4. Recurrence rate
Zhang (2001) [34]	88 (50:38)	8–56	40/48	6 months-12 years	(1) HMAI	(1) [mild] Cetirizine 10 mg PO qd [severe] Prednisone 5 mg PO tid (2) Ephedrine nasal drops 2–3 drops tid	6	1. effective rate
Group 4. Herbal integrated therapy vs. Western integrated therapy								
Xu et al. (2017) [35]	120 (60:60)	(A) 30.2 ± 9.1 (15–59) (B) 30.4 ± 9.2 (16–60)	(A) 32/28 (B) 31/29	T: 2.3 ± 0.4 (1–5) years C: 2.4 ± 0.5 (1–6) years	(1) Oral Xiangju Capsule 42 days (2) Xanthium powder HMAI 10 days	(A) Triamcinolone nasal spray 7 days (B) Loratadine 10 mg PO 7 days	(A) 42 (B) 7	1. Effective rate

IL, interleukin; NR, not reported; PO, per os (orally); tid, three times a day; qd, quaque die (every day); RQLQ, Rhinoconjunctivitis Quality of Life Questionnaire; TCM, Traditional Chinese Medicine; HM, Herbal Medicine; WM, Western Medicine; HMAI, Herbal Medicine Atomization Inhalation; TNF-α, tumor necrosis factor-alpha.

**Table 2:** Herbal medicine composition and atomization inhalation methods.

Study	Components of Herbal Medicine (Latin Name)	Treatment Duration/Time/Frequency	Method of Atomization Inhalation
Xiao et al. (2020) [22]	Armeniacae Semen Amarum, Peucedani Radix, Pogostemonis Herba, Platycodi Radix, Stemonae Radix 10 g, Xanthii Fructus 9 g, Magnoliae Flos, Cinnamomi Ramulus, Angelicae Dahuricae Radix, Glycyrrhizae Radix et Rhizoma 6 g, Ephedrae Herba 5 g	30 days/NR/qd	The herbs were ground, packed in bags, and heated using a traditional Chinese medicine steam machine. Patients were instructed to assume a suitable position and inhale the steam generated from the heated herbs. Care was taken to avoid burns, and patients were advised to inhale deeply to ensure that the steam fully entered the nasal cavity for optimal therapeutic effect.
Gong et al. (2022) [23]	Magnoliae Flos 12 g, Angelicae Dahuricae Radix 10 g, Xanthii Fructus 6 g, Menthae Herba 3 g, Coptidis Rhizoma 3 g.	14 days/NR/bid	The herbs were soaked in water, boiled, and filtered to obtain 10 mL of concentrated decoction, administered via an ultrasonic steam inhaler (WH-2000; Guangdong Yuehua Medical Equipment Factory).
Lu et al. (2020) [24]	Astragali Radix 25 g, Atractylodis Rhizoma Alba, Saposhnikoviae Radix, Paeoniae Radix, Zizyphi Fructus, Glycyrrhizae Radix et Rhizoma, Magnoliae Flos each 10 g, Xanthii Fructus 8 g, Cinnamomi Ramulus 6 g.	15 days/10–20 min/qod	The herbs were soaked in water for 30 min, brought to a boil over high heat, and simmered on low heat for 20 min. The resulting herbal liquid was poured into a thermos. Patients inhaled the steam for 10–20 min, with the nose positioned 15–25 cm away from the rim of the thermos.
Wang et al. (2014) [25]	Xanthii Fructus 42 g, Angelicae Dahuricae Radix 31 g, Chrysanthemi Flos 42 g, Acori Graminei Rhizoma 21 g, Rubiae Radix 31 g, Lonicerae Folium et Caulis 83 g.	60 days/NR/30 days qd and 30 days qod	In this method, 50 mL drug solution was diluted with 450 mL saline, and this solution and used as a nasal rinse once daily. After rinsing, ultrasonic nebulization was administered.
Lee (2014) [26]	Xanthii Fructus 100 g, Magnoliae Flos 100 g, Asiasari Radix et Rhizoma 50 g, Angelicae Dahuricae Radix 100 g, Menthae Herba 50 g.	14 days/30 min/bid	The herbs were ground to fine powder, divided into seven portions, and wrapped in a cloth, placed in a small-mouthed beaker, with hot water added until the herbs were submerged by a two-finger depth. The steam was inhaled for nasal treatment. Once cooled, the herbs were reheated to continue steaming.
Ye et al. (2022) [27]	Angelicae Dahuricae Radix 5.5 g, Magnoliae Flos 2.5 g, Xanthii Fructus 1.5 g, Menthae Herba 0.5 g.	7 days/15 min/qd	The herbs were ground into powder and placed in a non-woven fabric bag. The herb bag was placed in a small container, and boiling water (100 °C) was added until the herbs were submerged by 5 cm. The patient was seated approximately 15 cm away from the container, allowing the steam to treat the nasal cavity.
Zhang et al. (2012) [28]	Magnoliae Flos, Angelicae Dahuricae Radix, Angelicae Sinensis Radix, Houttuyniae Herba.	7 days/15 min/qd or bid	In this method, 10 mL Xin Ju nebulization solution (formulated by the hospital’s pharmacy) was mixed with 10 mL saline and administered via ultrasonic nebulization.
Xiulan (2021) [30]	Inulae Radix, Sophorae Radix, Rubus Coreanus Stem, Kaempferiae Rhizoma.	21 days/15–20 min/bid	In this method, 3–5 g of Siwei Tumuxiang Decoction was poured into a stainless-steel cup with 5–7 cm diameter. After adding 150–200 mL of water, it was simmered over low heat until it boils (approximately 2 min). A wet towel was wrapped around the cup’s rim, and the patient placed their mouth and nose over the cup and inhaled the steam deeply for 5–7 min, while covering their mouth and nose with the towel. When the temperature of the decoction dropped to approximately 50 °C, the decoction was reheated, and the steaming process was repeated thrice for approximately 15–20 min. After the steam treatment, the decoction was consumed.
Gao (2014) [31]	Platycodonis Radix, Scrophulariae Radix, Pinelliae Tuber, Zingiberis Rhizoma Recens, Poria Sclerotium, Citri Unshius Pericarpium, Armeniacae Semen, Astragali Radix, Atractylodis Rhizoma Alba, Saposhnikoviae Radix, Glycyrrhizae Radix et Rhizoma.	7 days/10 min/qd	The herbs were processed in the laboratory using modern equipment to create a brown liquid of high purity and effective concentration, in 100 mL batches, and stored at −20 °C for future use. The treatment group received 20 mL of this liquid for nebulized inhalation.
Zeng et al. (2012) [32]	Xanthii Fructus 10 g, Magnoliae Flos 5 g, Angelicae Dahuricae Radix 10 g, Scutellarie Radix 10 g, Astragali Radix 20 g, Menthae Herba 5 g, Cnidii Rhizoma 5 g, Fritillariae Thunbergii Bulbus 10 g, Glycyrrhizae Radix et Rhizoma 5 g, Chrysanthemi Flos 10 g, Glycine Semen Preparata 10 g.	21 days/10 min/qd	20 mL of Cang Xin solution via ultrasonic nebulization
Chen (2012) [33]	Ephedrae Herba 9 g, Cinnamomi Ramulus 6 g, Asiasari Radix et Rhizoma 3 g, Paeoniae Radix 9 g, Zingiberis Rhizoma 3 g, Pinelliae Tuber 9 g, Schisandrae Fructus 3 g, Glycyrrhizae Radix et Rhizoma 6 g.	21 days/NR/bid	The decoction was prepared once a day, boiled twice, and divided into two doses for morning and evening use. After preparing the decoction, the residue was re-boiled with water, used for nasal steaming using a small cup, and applied to the nasal area twice a day.
Zhang (2001) [34]	Xanthii Fructus, Magnoliae Flos, Angelicae Dahuricae Radix, and Menthae Herba each 10 g, Asiasari Radix et Rhizoma 3 g, Astragali Radix 30 g, Atractylodis Rhizoma Alba, Saposhnikoviae Radix each 15 g.	6 days/30 min/bid	The herbal ingredients were soaked and decocted for 30 min. Approximately 100 mL of the decoction was filtered to remove the residue, and after cooling, the liquid was placed in the nebulizer. The liquid was atomized into fine particles, and the patient wore a mask over their mouth and nose while performing slightly deeper breathing exercises.
Xu et al. (2017) [35]	Angelicae Dahuricae Radix, Magnoliae Flos, Xanthii Fructus, and Menthae Herba each 10 g, Asiasari Radix et Rhizoma 3 g.	10 days/30 min/bid	The herbs were soaked in clean water for 30 min, and boiled gently with water for approximately 30 min. The resulting liquid (100 mL) was collected, sedimented to remove residue, and allowed to cool. The liquid was subsequently placed in a nebulizer bottle for ultrasonic nebulization therapy. During treatment, patients were instructed to perform slightly deeper breathing exercises.

bid, bis in die (twice a day); NR, not reported; qd, quaque die (once a day); qod, quaque altera die (every other day).

**Table 3:** Frequency of herbal medicines used in the included studies.

Herbal Medicines (Latin Names)	Frequency of Use
Xanthii Fructus, Magnoliae Flos	10
Angelicae Dahuricae Radix	9
Menthae Herba, Glycyrrhizae Radix et Rhizoma	6
Astragali Radix	5
Asiasari Radix et Rhizoma, Cinnamomi Ramulus, Saposhnikoviae Radix, Atractylodis Rhizoma Alba	4
Paeoniae Radix	3
Ephedrae Herba, Armeniacae Semen, Chrysanthemi Flos, Pinelliae Tuber, Platycodonis Radix, Zizyphi Fructus	2
Rubiae Radix, Pogostemonis Herba, Acori Graminei Rhizoma, Peucedani Radix, Fritillariae Thunbergii Bulbus, Lonicerae Folium et Caulis, Citri Unshius Pericarpium, Cnidii Rhizoma, Poria Sclerotium, Coptidis Rhizoma, Zingiberis Rhizoma Recens, Zingiberis Rhizoma, Stemonae Radix, Glycine Semen Preparata, Scrophulariae Radix, Inulae Radix, Rubus idaeus L., Sophorae Radix, Kaempferiae Rhizoma, Angelicae Sinensis Radix, Houttuyniae Herba, Scutellariae Radix, Schisandrae Fructus	1

**Table 4:** GRADE assessment for the meta-analysis of the included studies.

Certainty Assessment							No. of Patients		Effect		Certainty of Evidence
No. of Studies	Study Design	Risk of Bias	Inconsistency	Indirectness	Imprecision	Other Considerations	HMAI	Control	Relative (95% CI)	Absolute (95% CI)	
Group 1											
5	Randomized control trials	serious ^a^	not serious	not serious	not serious	none	260/284 (91.5%)	220/284 (77.5%)	RR: 1.18 (1.10–1.27)	139 more per 1000 (77 more to 209 more)	⨁⨁⨁◯ Moderate ^a^
Group 2											
3	Randomized control trials	serious ^a^	serious^b^	not serious	not serious	none	181/205 (88.3%)	123/185 (66.5%)	RR: 1.33 (1.07–1.66)	219 more per 1000 (47 more to 439 more)	⨁⨁◯◯ Low ^a,b^
Group 3											
5	Randomized control trials	serious ^a^	serious ^b^	serious ^c^	not serious	none	260/272 (95.6%)	199/256 (77.7%)	RR: 1.21 (1.10–1.33)	163 more per 1000 (78 more to 257 more)	⨁◯◯◯ Very low ^a,b,c^

CI, confidence interval; RR: risk ratio; HMAI: Herbal Medicine Atomization Inhalation; GRADE, Grading of Recommendations Assessment, Development, and Evaluation. ^a^ Most studies were assessed for high risk of bias. ^b^ Substantial heterogeneity was assessed. ^c^ Some interventions include oral administration of the decoctions that are used in HMAI.

## Data Availability

No new data were created or analyzed in this study. Data sharing is not applicable to this article.

## Supplementary Materials

The following supporting information can be downloaded at: https://www.mdpi.com/article/10.3390/ph18121877/s1, File S1: PRISMA checklist; File S2: Search terms used in each database.

## Abbreviations

AR	Allergic Rhinitis
CI	Confidence Interval
FEM	Fixed-effect Model
GRADE	Grading of Recommendations Assessment, Development, and Evaluation
HM	Herbal Medicine
HMAI	Herbal medicine atomization inhalation
Ig	Immunoglobulin
IL	Interleukin
INSs	Intranasal corticosteroids
NF-κB	Nuclear Factor kappa-light-chain-enhancer of activated B cells
PRISMA	Preferred Reporting Items for Systematic Reviews and Meta-Analyses
RCT	Randomized Controlled Trial
REM	Random-effect Model
RoB	Cochrane Risk of Bias tool
RQLQ	Rhinoconjunctivitis Quality of Life Questionnaire
RR	Risk Ratio
TCM	Traditional Chinese Medicine
TER	Total Effective Rate
TNF-α	Tumor necrosis factor-alpha
WM	Western medicine
