# Supplementary material for: Herbal Inhalation Therapy for Allergic Rhinitis: A Systematic Review and Meta-Analysis
Source: Pharmaceuticals (Basel). 2025 Dec 11;18(12):1877. doi: 10.3390/ph18121877 (PMC12736282; doi:10.3390/ph18121877)
Supplement: Supplementary file 1 [file pharmaceuticals-18-01877-s001.zip › Supplementary_File_S2.pdf]

Supplementary File S2

Search terms used in each database

Medline (via PubMed)

	**Search terms**	**Results**
1	hinitis [Mesh] OR rhiniti* [TW] OR rhinosinusitis [TW] OR rhinoconjunctivitis [TW] OR ozena* [TW]	2,194
2	umigation [Mesh] OR fumigations [TW] OR steam [TW] OR atomization [TW]	7,291
3	nhalation [Mesh] OR Inhaling [TW] OR “Inspiration, Respiratory” [TW] OR “Respiratory Inspiration”[TW] OR Aroma*[TW]	76,726
4	romatherapy [Mesh] OR Aromatherapies [TW] OR “Aroma Therap*” [TW]	,116
5	Nebulizers and Vaporizers” [Mesh] OR Vaporizer* [TW] OR Inhaler* [TW] OR Inhalator* [TW] OR Nebulizer* [TW] OR Atomizer* [TW] OR “Inhalation Device*” [TW] OR “Devices, Inhalation” [TW]	2,948
6	(“Controlled Clinical Trials as Topic” [Mesh] OR “Randomized Controlled Trials as Topic” [Mesh] OR Randomized Controlled Trial [PT] OR Controlled Clinical Trial [PT] OR Multicenter Study [PT] OR “OR randomised [TW] OR (Double*[TW] OR single*[TW] OR treb* [TW] OR tripl* [TW]) AND (Blind* [TW] OR mask* [TW])) OR controlled-clinical-trial* [TW] OR controlled-trial* [TW] OR placebo* [TW] OR randomly* [TW]) NOT (Case Reports [PT] OR Letter [PT] OR meta-analys* [TW] OR metaanalys* [TW] OR “Meta-Analysis” [PT] OR case-report* [TW] OR Letter* [TI] OR “Systematic Review” [PT] OR Systematic-Review* [TI]))	,754,546
7	1 AND (#2 OR #3 OR #4 OR #5) AND #6	52

EMBASE (via Elsevier)

	Search terms	Results
1	Rhinitis'/exp OR rhinitis OR rhiniti*:ab,ti,kw OR rhinopath:ab,ti,kw OR rhinosinusit:ab,ti,kw OR rhinoconjunctivitis:ab,ti,kw OR ozena*:ab,ti,kw	28,631
2	fumigation'/exp OR fumigations:ab,ti,kw OR steam:ab,ti,kw OR atomization:ab,ti,kw	8,057
3	inhalation'/exp OR inhaling:ab,ti,kw OR 'inspiration, respiratory':ab,ti,kw OR 'respiratory inspiration':ab,ti,kw OR 'inhaled material':ab,ti,kw OR 'inhaled particle':ab,ti,kw	3,187
4	aroma'/exp	,243
5	nebulizers'/exp OR 'aero comfort':ab,ti,kw OR 'aero mist':ab,ti,kw OR 'erapid':ab,ti,kw OR 'innospire':ab,ti,kw OR 'innospire deluxe':ab,ti,kw OR 'innospire essence':ab,ti,kw OR 'innospire go':ab,ti,kw OR 'innospire mini':ab,ti,kw OR 'nebuliser':ab,ti,kw OR 'nebuliser (physical object)':ab,ti,kw OR 'nebuliser each':ab,ti,kw OR 'nebuliser misc. each':ab,ti,kw OR 'nebulisers and vapourisers':ab,ti,kw OR 'nebulization equipment':ab,ti,kw OR 'nebulizator':ab,ti,kw OR 'nebulizer (physical object)':ab,ti,kw OR 'nebulizer each':ab,ti,kw OR 'nebulizer misc. each':ab,ti,kw OR 'nembulizer':ab,ti,kw OR 'sidestream (nebulizer)':ab,ti,kw OR 'sidestream pediatric':ab,ti,kw OR 'sidestream plus':ab,ti,kw	4,358
6	vaporizers'/exp OR 'emo vaporizer':ab,ti,kw OR 'emo vapouriser':ab,ti,kw OR 'isotec':ab,ti,kw OR 'vapor 19':ab,ti,kw OR 'vaporiser':ab,ti,kw OR 'vaporizer each':ab,ti,kw OR 'vaporizer misc. each':ab,ti,kw OR 'vaporizers':ab,ti,kw OR 'vapour 19':ab,ti,kw OR 'vapouriser':ab,ti,kw OR 'vapouriser each':ab,ti,kw OR 'vapouriser misc. each':ab,ti,kw OR 'vapourisers':ab,ti,kw OR 'vaporizer':ab,ti,kw	,943
7	randomized controlled trial'/exp OR [randomized controlled trial]/lim OR 'controlled clinical trial'/de OR 'multicenter study'/exp OR 'randomization'/exp OR 'random allocat*':ab,ti,kw OR randomized:ab,ti,kw OR randomised:ab,ti,kw OR 'controlled clinical trial*':ab,ti,kw OR 'controlled trial*':ab,ti,kw OR placebo*:ab,ti,kw OR randomly*:ab,ti,kw OR (((double* OR single* OR treb* OR tripl*) NEAR/3 (blind* OR mask*)):ab,ti,kw)) NOT ([conference abstract]/lim OR [conference paper]/lim OR [conference review]/lim OR [data papers]/lim OR	,488,183

	[editorial]/lim OR [erratum]/lim OR [letter]/lim OR [note]/lim OR [review]/lim)	
8	1 AND (#2 OR #3 OR #4 OR #5 OR #6) AND #7	22

CENTRAL

	Search terms	Results
1	mh rhinitis] OR (rhiniti* OR rhinopath OR rhinosinusit OR rhinoconjunctivitis OR ozena*):ab,ti,kw	1,789
2	mh fumigation] OR (Fumigations OR steam OR atomization):ab,ti,kw	49
3	nhalation OR Inhaling OR “Inspiration, Respiratory” OR “Respiratory Inspiration” OR “inhaled material” OR “inhaled particle”):ab,ti,kw	3,644
4	mh aromatherapy] OR (aromatherapies OR ″aroma Therap*” OR “Therap*, aroma”):ab,ti,kw	03
5	mh “Nebulizers and Vaporizers”] OR (Vaporizer* OR Atomizers OR “Device*, Inhalation” OR “Inhalation Device*” OR Nebulizer* OR “Vaporizers and Nebulizers” OR Inhaler*):ab,ti,kw	0,053
6	1 AND (#2 OR #3 OR #4 OR #5)	22
